# Stereotactic radiosurgery for intraventricular meningioma: a systematic review and meta-analysis

**DOI:** 10.1007/s00701-024-06185-w

**Published:** 2024-07-09

**Authors:** Alireza Soltani Khaboushan, Mohammad Amin Dabbagh Ohadi, Hanieh Amani, Mohammad Dashtkoohi, Arad Iranmehr, Jason P. Sheehan

**Affiliations:** 1 School of Medicine, Tehran University of Medical Sciences, Tehran, Iran; 2 Students’ Scientific Research Center, Tehran University of Medical Sciences, Tehran, Iran; 3 Neurosurgery Department, Imam Khomeini Hospital Complex (IKHC), Tehran University of Medical Sciences, Tehran, Iran; 4 Student Research Committee, Faculty of Medicine, Mashhad University of Medical Sciences, Mashhad, Iran; 5 Department of Neurological Surgery, University of Virginia, Charlottesville, VA USA

**Keywords:** Radiosurgery, Meningioma, Progression-free survival, Brain edema

## Abstract

**Background:**

Intraventricular meningioma (IVM) is a rare subtype of intracranial meningioma, accounting for 9.8 to 14% of all intraventricular tumors. Currently, there is no clear consensus on which patients with IVM should receive conservative treatment, surgery, or stereotactic radiosurgery (SRS). This research aims to analyze the outcomes, including survival and recurrence rates of patients who undergo SRS for IVM as a primary or adjuvant treatment.

**Methods:**

A systematic search was conducted in Scopus, Web of Science, PubMed, and Embase till June 5th 2023. Screening and data extraction were performed by two independent authors. Random-effect meta-analysis was performed to determine the tumor control proportion of IVM cases treated with SRS. Individual patient data (IPD) meta-analysis was performed for the progression-free survival (PFS) of the patients in the follow-up time. All analyses were performed using the R programming language.

**Results:**

Out of the overall 132 records, 14 were included in our study, of which only 7 had enough data for the meta-analysis. The tumor control proportion was 0.92 (95% CI, 0.69–0.98) in patients who underwent SRS for primary IVM. The overall tumor control in both primary and adjuvant cases was 0.87 (95% CI, 0.34–0.99). the heterogeneity was not significant in both meta-analyses (*P* = 0.73 and *P* = 0.92, respectively). Post-SRS perifocal edema occurred in 16 out of 71 cases (0.16; 95% CI, 0.03–0.56), with no significant heterogeneity (*P* = 0.32). IPD meta-analysis showed a PFS of 94.70% in a 2-year follow-up. Log-rank test showed better PFS in primary SRS compared to adjuvant SRS (*P* < 0.01).

**Conclusions:**

According to this study, patients with IVM can achieve high rates of tumor control with a low risk of complications when treated with SRS, regardless of whether they have received prior treatment. Although SRS could be a promising first-line treatment option for asymptomatic IVM, its efficacy in symptomatic patients and its comparison with resection require further investigation.

**Supplementary Information:**

The online version contains supplementary material available at 10.1007/s00701-024-06185-w.

## Introduction

Intraventricular meningioma (IVM) accounts for 9.8 to 14% of all intraventricular tumors [39] and is a rare subtype of intracranial meningioma, which comprises 0.5% to 3.7% of all meningiomas [8]. This type of meningioma is a unique entity compared to other subtypes in terms of its epidemiology and pathology [28, 31, 38, 39]. The prevalence of meningiomas increases with age [17, 25]. According to a systematic review, most IVMs occur in middle-aged adults with a mean age of 42.2 years [39]. Furthermore, while meningiomas are more frequently diagnosed in women [4], this gender bias is less pronounced in cases of IVM [39]. Individuals who are afflicted with IVM are likely to manifest symptoms at an earlier stage owing to the tumor’s compressive effect on crucial structures or heightened intracranial pressure. These symptoms may include headaches, impaired vision, defects in the visual field, memory impairment, or seizures [3, 28, 31, 35, 38]. Although most IVMs are classified as Grade I according to the WHO 2016 classification [14, 21, 24], studies have shown a higher prevalence of fibrous subtypes in IVMs, which are more aggressive and have a higher rate of relapse [39].

Gross total resection is often curative due to the mostly benign nature of these tumors, but their location in close proximity to important structures, such as the visual pathway and venous sinuses, can pose a challenge for resection [12, 39]. For patients who are not suitable for general anesthesia or refuse surgery, stereotactic radiosurgery (SRS) may be recommended as an alternative option [27]. Nevertheless, the intraventricular location of these tumors comes with certain limitations for this technique [27]. Also, SRS may carry risks of complications such as adverse radiation effects, particularly for larger tumors and higher doses of radiation, and primary SRS for IVM seems to be associated with a higher chance of peritumoral edema [19, 37]. In such cases, patients may require steroid treatment or even neurosurgical intervention [8].

Currently, there is no definitive agreement regarding which patients with IVM are suitable for conservative treatment, resection, or SRS [8]. The purpose of this research is to gain a deeper understanding of the outcomes of patients who receive SRS for IVM and to analyze survival rates and recurrence rates in patients who underwent SRS as the initial treatment and those who underwent SRS for recurrent or residual disease.

## Methods

### Search strategy and databases

For this systematic review, Medline, Embase, Scopus, and Web of Science were searched without any date and type of study limits to retrieve relevant studies. The complete search string for each database and the number of results is available as Supplementary Material 1. The search result was updated until June 5th, 2023. This review is reported in accordance with Preferred Recording Items for Systematic Review and Meta-Analysis (PRISMA). Also, the bibliography of the included articles will be searched for potentially relevant papers. The protocol of the study has been registered in the PROSPERO (https://www.crd.york.ac.uk/prospero/display_record.php?ID=CRD42023452783) with the registration ID of CRD42023452783.

### Eligibility criteria

Studies that applied SRS for the resection of IVM were eligible for the review. Studies were included if they met the inclusion criteria: 1) original studies on human subjects, 2) diagnosis of isolated IVM, 3) application of stereotactic radiosurgery for treatment of IVM, and 4) providing the follow-up outcome of the patients. Studies were excluded if they had the following exclusion criteria: 1) reviews, letters, and book chapters, 2) in vivo and in vitro studies, 3) Other diseases or complications interfering with meningioma, and 4) studies without sufficient follow-up and data.

### Screening and study selection

After the removal of the duplicate articles, two independent authors (ASK and HA) screened the results retrieved from databases. Discrepancies were resolved by discussion.

### Data extraction

Two authors (ASK and HA) independently extracted the data. The following data were extracted from the studies: the first author’s name, publication year, location of study, total number of cases, number of primary SRS cases, mean age, male percentage, presentation symptoms, history of previous surgery, mean tumor volume, tumor location, marginal and maximum dosage of radiation, mean follow-up time, overall progression-free survival (PFS), complications of the treatment and adverse events, including perifocal edema. In cases where individual data were available, the aforementioned data were extracted for individual participant data (IPD) meta-analysis.

To gather the missing data, we reached out to the corresponding authors. For any data presented in figures and plots, we utilized WebPlotDigitizer (https://apps.automeris.io/wpd/) to extract the necessary information.

### Quality assessment

The quality of the included studies was critically assessed by two independent reviewers (ASK and HA). Joanna Briggs Institute’s (JBI) checklist for case series and case reports was used to appraise the quality of the included studies [32]. The JBI checklist contains 10 questions for case series studies and 8 questions for case reports. Incongruences were resolved by discussion. Each item is answered with “yes,” “no,” “unclear,” and “not applicable.” We considered a high risk of bias if “yes” answers were ≤ 50% and a low risk of bias if “yes” answers were higher than 50%.

### Statistical analysis

Data were collected as the number of progression-free of IVM tumors through the follow-up and the total number of tumors. The proportion of the progression-free tumors to the total number of tumors was used as the effect. The general linear mixed model method was used to perform the meta-analysis on logit-transformed proportions, and Clopper-Pearson was used to estimate the confidence interval for studies. We only considered studies with more than 3 tumors in the meta-analysis; thus, only case-series were included for the meta-analysis. Moreover, a meta-analysis was performed to determine whether edema was symptomatic or asymptomatic following SRS. *P*-values under 0.05 were considered statistically significant. The analyses were performed for primary SRS and overall (primary and adjuvant) SRS applications, and their results were compared. Egger’s test was used to evaluate the funnel plot asymmetry to assess publication bias. The trim and fill method was used to adjust the effect size if there was significant publication bias. Cochran’s Q test and I2 were employed to determine the presence of heterogeneity, with a *P*-value of lower than 0.10, indicating statistically significant heterogeneity. *I*^2^ heterogeneity levels were categorized as low (≤ 25%), moderate (25–75%), or high (≥ 75%). For sensitivity analysis, a leave-one-out analysis was performed, through which each time, one study was omitted, and the effect was calculated to assess if it was placed within the overall 95% CI. Moreover, meta-regression was performed for median tumor volume (cc), mean age of participants, median marginal dose of SRS, and year of publication, where the data were available. For IPD, follow-up times and occurrence of the events for primary and non-primary SRS were recorded to fit the Cox proportional hazard regression model. The comparison was performed to capture the difference between tumor control in primary SRS and adjuvant SRS. All analyses were performed using “meta,” “metafor,” and “survival” packages (R programming language version 4.2.1).

## Results

### Study selection and characteristics

**Fig. 1:**
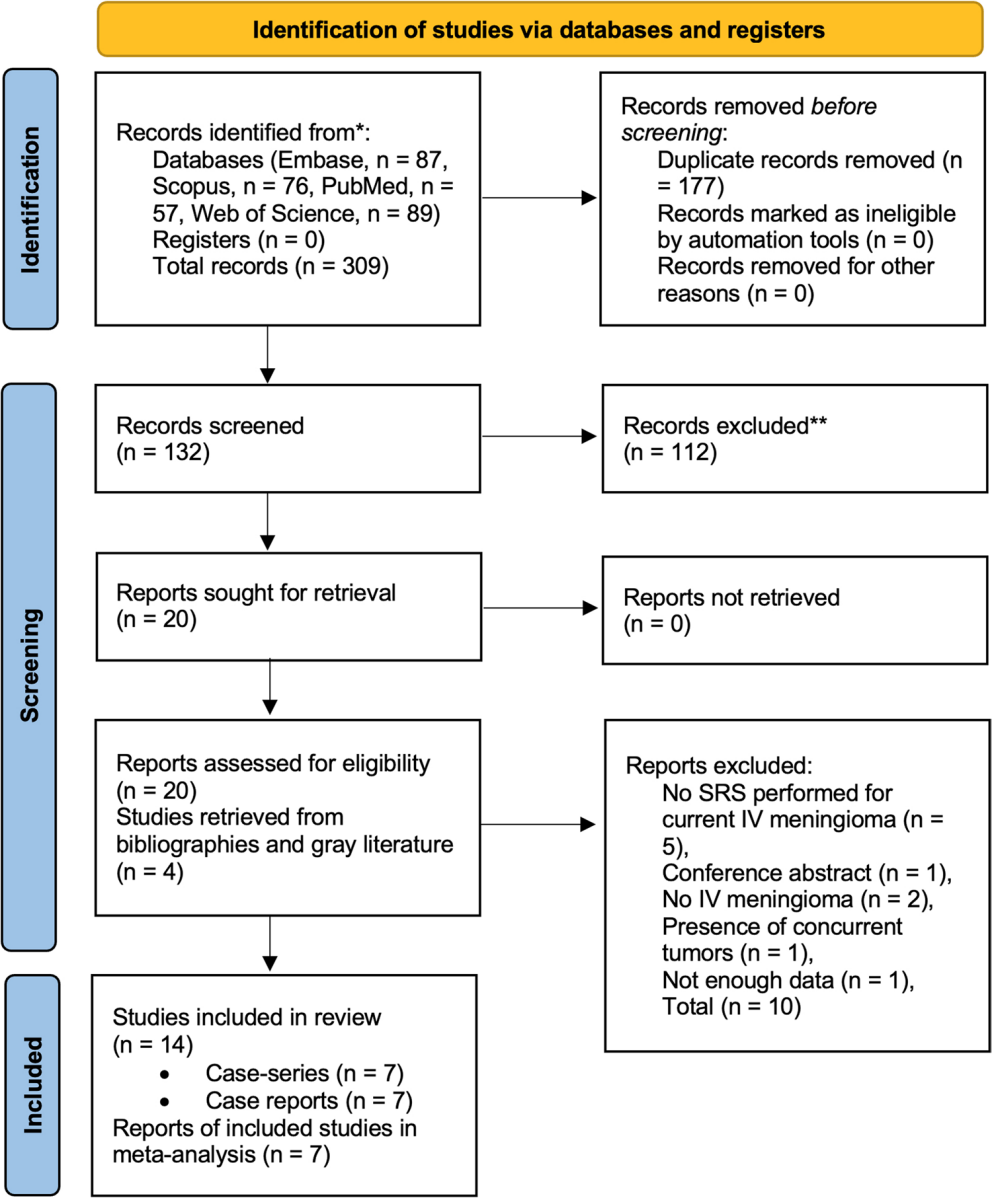
Shows the PRISMA flow diagram for the screening process of the papers

Search retrieved 309 articles from Medline, Scopus, Embase, and Web of Science. After duplicate removal, 132 papers remained, of which 20 were included after the title/abstract screening stage [1, 8, 10, 11, 15, 16, 18, 20, 22, 23, 29, 30, 36, 37, 40–42, 45, 46, 48] and 4 were retrieved from the bibliography review [2, 6, 33, 43]. In the full-text review, 10 articles did not meet our inclusion criteria [8, 11, 20, 29, 37, 41, 42, 45, 48]. Among excluded articles, 5 did not apply SRS for the treatment of current IVM [16, 22, 30, 36, 40], 2 did not have patients with IVM [11, 18], one was conference abstract [45], one did not have enough data [2], and one had multiple spinal hemangioblastomas along with IVM and without application of SRS [1]. Overall, 14 articles matched our inclusion criteria [6, 8, 10, 15, 20, 23, 29, 33, 37, 41–43, 46, 48]. Figure 1 shows the PRISMA flow diagram for the screening process of the papers.

A total 14 studies and 101 tumors were included, which comprise, seven case-series [8, 10, 20, 29, 41, 43, 48] and seven case reports [6, 15, 23, 33, 37, 42, 46], and only seven [8, 10, 20, 29, 41, 43, 48] had enough data with at least 3 patients to be entered into the meta-analysis. If studies, case reports or case series, had sufficient data, they were also included in the individual patient tumor control analysis. There were no fractioned SRS among the studies and all used single-session procedure. A summary of case reports was only reported in the study characteristics table (Table 1). The primary and total tumors that underwent SRS with available follow-up were used for meta-analysis. A summary of study characteristics is available in Table 1.

**Table 1:** Summary of characteristics of included studies

Study	Pt. no (Primary /Total tumor number)	Female	Mean Age (years)	Median Volume (ml)	Tumor location	Tumor grade and histopathology	Complications	Median Margin Dose, Gy (Range)	Median Follow-Up Months (Range)
Terada, 1993	1 (0/1)	100%	58	40 mm diameter	Lateral ventricle 100%	NA	No reported complications	12	24
Ide, 2004	1 (0/1)	100%	24	NA	Lateral ventricle 100%	Grade II: 100%	Memory disturbance and lethargy	16	63
Kim, 2009	9 (5/9)	33%	51 (14–81)	3.9	Trigone: 89% 3th Ven: 11%	Grade I: 83.3% Grade II: 16.7%	No reported complications	16 (14–25)	64 (7–161)
Nundkumar, 2013	2 (2/2)	100%	50 (49–50)	3.3	Trigone 100%	Grade I 100%	Peritumoral edema (100%)	18 (18–18)	12 (8–17)
Chen, 2013	1 (0/1)	100%	41	NA	Trigone 100%	NA	No reported complications	16 to 81% Isodose line	36
Nanda, 2016	1 (1/1)	NA	NA	NA	Lateral ventricle 100%	NA	NA	NA	6
Wang, 2018	1 (0/1)	0%	37	NA	3th Ven: 100%	NA	NA	NA	6
Liu, 2019	1 (0/1)	100%	52	NA	Lateral ventricle 100%	NA	Headache and progressive paralysis of the left limb	16 to 50% Isodose curve	2
Mindermann, 2020	5 (6/6)	100%	63 (50–81)	4.7 (2.5–14.1)	Trigone 100%	NA	Perifocal edema (80%)	13.5 (12–15)	18 (19–240)
Samanci, 2020	6 (5/6)	66%	Median 41 (30–71)	5.45	Lateral Ven: 100%	NA	Tumor enlargement (17%), perifocal edema (17%)	12 (11–13)	74 (24–139)
Christ, 2023	33 (33/33)	73%	Median 58 (20–71)	6.7	Trigone: 76% Posterior horn: 9% lateral Ven: 6% 4th ventricle 3%	NA	No reported complications	14 (12–16)	73.2 (21.6- 214.8)
Daza-Ovalle, 2023	19 (14/20)	37%	Median 53 (14–84)	4.8	Trigone: 90%, 3th Ven: 5%, 4th Ven:5%	Grade I: 45% Grade II: 5% Unknown: 50%	Perifocal edema (37%)	14 (12–25)	63.1 (6–322.4)
Umekawa, 2023	11 (12/12)	55%	Median 45 (13–80)	4.9	Trigon: 75%, lateral Ven: 17%, 3th Ven: 8%	NA	Headache (8%), perifocal edema (37%)	16 (9–18)	52 (3–353)
Yu, 2023	7 (0/7)	NA	NA	NA	NA	Grade I: 100%	NA	NA	NA

### Quality assessment

Using the JBI tool for the quality assessment of case series [8, 10, 20, 29, 41, 43, 48], 6 out of 7 had high-quality [8, 20, 29, 41, 43, 48], and one had moderate quality [10]. Among case reports, 6 out of 7 had high-quality [6, 15, 23, 37, 42, 46], and one had low-quality [33]. The summary report of quality assessments for case-series and case reports is available in Tables 2 and 3, respectively.

**Table 2:** Quality assessment for case series studies

Reference	Q1	Q2	Q3	Q4	Q5	Q6	Q7	Q8	Q9	Q10	percentage
Umekawa, M. et al	yes	yes	yes	yes	yes	yes	yes	yes	yes	yes	100
Daza-Ovalle, A. et al	unclear	yes	yes	unclear	no	yes	yes	yes	yes	yes	40
Christ, S. M. et al	unclear	yes	yes	unclear	no	yes	yes	yes	yes	yes	80
Samanci, Y. et al	yes	yes	yes	yes	yes	yes	yes	yes	yes	yes	100
Mindermann, T. et al	yes	yes	yes	yes	yes	yes	yes	yes	yes	yes	100
Kim I. Y. et al	no	yes	yes	yes	yes	yes	yes	yes	yes	yes	90
Yu, J. et al	yes	yes	yes	no	yes	yes	yes	yes	yes	yes	90

Q1: Were there clear criteria for inclusion in the case series? Q2: Was the condition measured in a standard, reliable way for all participants included in the case series? Q3: Were valid methods used for identification of the condition for all participants included in the case series? Q4: Did the case series have consecutive inclusion of participants? Q5: Did the case series have complete inclusion of participants? Q6: Was there clear reporting of the demographics of the participants in the study? Q7: Was there clear reporting of clinical information of the participants? Q8: Were the outcomes or follow-up results of cases clearly reported? Q9: Was there clear reporting of the presenting site(s)/clinic(s) demographic information? Q10: Was statistical analysis appropriate?

**Table 3:** Quality assessment for case report studies

Author	Q1	Q2	Q3	Q4	Q5	Q6	Q7	Q8	Percentage
Nanda, A. et al	unclear	no	no	yes	yes	no	no	no	25
Nundkumar N. et al	yes	yes	yes	yes	yes	yes	yes	yes	100
Terada, T. et al	yes	yes	yes	yes	yes	yes	yes	yes	100
Liu, J. et al	yes	yes	yes	no	yes	yes	unclear	yes	75
Chen, C. C. et al	yes	yes	yes	yes	yes	yes	unclear	yes	87.5
Ide, M. et al	yes	yes	yes	yes	yes	yes	yes	yes	100
Wang, Y. et al	yes	no	yes	yes	yes	yes	unclear	yes	75

Q1: Were patient’s demographic characteristics clearly described? Q2: Was the patient’s history clearly described and presented as a timeline? Q3: Was the current clinical condition of the patient on presentation clearly described? Q4: Were diagnostic tests or assessment methods and the results clearly described? Q5: Was the intervention(s) or treatment procedure(s) clearly described? Q6: Was the post-intervention clinical condition clearly described? Q7: Were adverse events (harms) or unanticipated events identified and described? Q8: Does the case report provide takeaway lessons?

### Tumor progression control

**Fig. 2:**
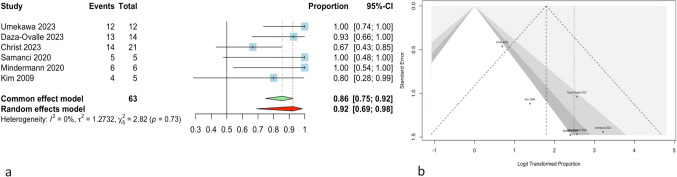
**a** The forest plot for the meta-analysis shows 92% overall tumor control in cases that underwent primary SRS for IVM. Also, the heterogeneity of studies was not significant (*P* = 0.73). **b** The funnel plot shows that there is asymmetry, showing the potential publication bias among the studies

Successful tumor control was reported in 54 out of the 63 primary tumors among 61 patients who underwent SRS as a first-line treatment, with a proportion of 0.92 (95% CI, 0.69–0.98). The forest plot and Egger’s linear regression test for asymmetry of the funnel plot are depicted in Fig. 2 (*P* < 0.05). The overall heterogeneity of the studies was not statistically significant (*Q*: 2.82, *P*-value: 0.73, tau^2^: 1.27, and *I*^2^: 0%). Due to publication bias, the trim-and-fill method was used, resulting in a 0.73 overall proportion with 3 added studies (95% CI, 0.54–0.87; tau^2^, 0.45; *I*^2^, 43.1%). Leave-one-out analysis demonstrated that omitting the Christ and Daza-Ovalle studies [8, 10] could considerably affect the overall effect of the analysis. Influence diagnostics are shown in Supplementary Fig. 1.

**Fig. 3:**
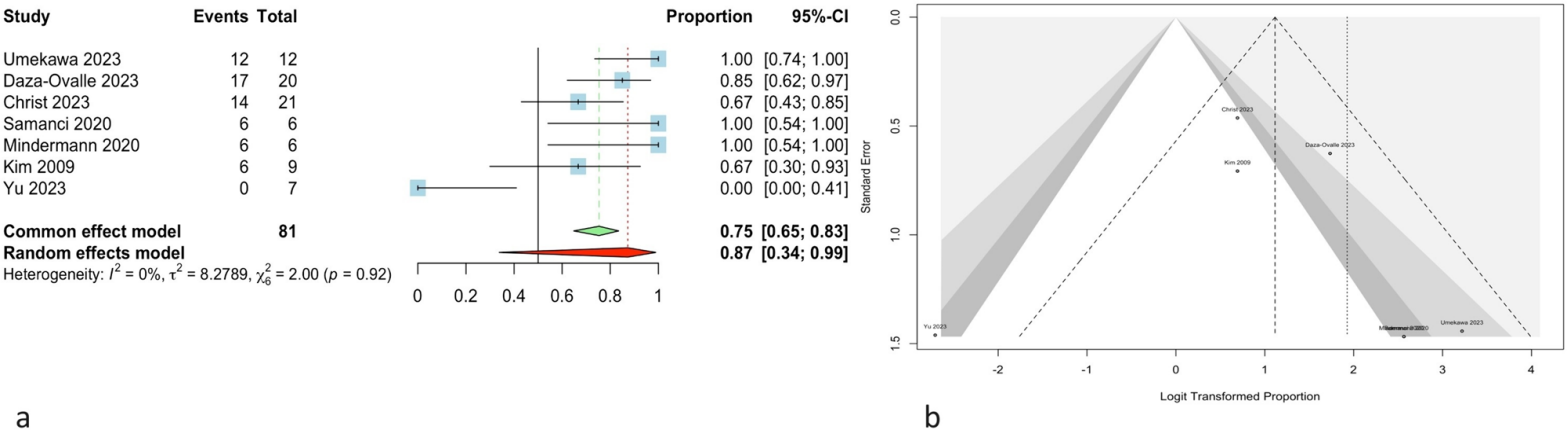
**a** The forest plot for the meta-analysis shows 87% overall tumor control in cases that underwent SRS for IVM, regardless of the type of SRS (primary or non-primary). Also, the heterogeneity of studies was not significant (*P* = 0.92). **b** The funnel plot indicates no significant asymmetry, suggesting no publication bias among the studies

Assessing the overall impact of SRS on IVM, we observed successful tumor control in 61 out of 81 tumors among 79 patients during the follow-up period, yielding a proportion of 0.87 (95% CI, 0.34–0.99). The forest plot and Egger’s linear regression test for asymmetry of the funnel plot are depicted in Fig. 3 (*P* = 0.69). The heterogeneity of the studies was low (*Q*: 2.00; *P*-value: 0.92, tau^2^: 8.28, and *I*^2^: 0%). After conducting a leave-one-out analysis, it was found that excluding the Christ and Daza-Ovalle studies [8, 10] could have a significant impact on the overall analysis. Omitting other studies did not change the overall effect significantly. Influence diagnostics of the studies have been depicted in Supplementary Fig. 2. Furthermore, comparing the primary and adjuvant SRS effects demonstrated significantly higher tumor control in primary SRS (*P* < 0.05).

Meta-analysis for assessment of the tumor shrinkage was performed in 4 studies [10, 20, 41, 43]. Overall, 27 tumors regressed after SRS out of a total of 41 tumors with a proportion of 0.66 (95% CI, 0.50–0.79). The forest plot and funnel plot of the meta-analysis are shown in Supplementary Fig. 3. The overall heterogeneity was not significant (*Q*: 0.13, *P*-value: 0.99, tau^2^: 0, and *I*^2^: 0%). Egger linear regression for publication bias was not significant (*P*-value: 0.06). No outlier was detected based on the sensitivity analysis.

Meta-regression for median tumor volume, mean age of participants, median marginal radiation dosage, and year of publication were performed. It was demonstrated that none of these variables could significantly affect the tumor progression in primary SRS for IVM. The *P*-values for tumor volume, age, marginal dose, and year of the study were 0.99, 0.91, 0.77, and 0.76, respectively. However, we found higher median volume (effect = 2.07, *P* < 0.05) and median marginal dose (effect = 0.19, *P* < 0.05) associated with better tumor control in the pooled cohort of all tumors. Meta-regression for the mean age of participants and publication year did not have significant results, with *P*-values of 0.74 and 0.97, respectively. The summary results of the regression are shown in Table 4.

**Table 4:** Summary of meta-regressions

Analysis	Covariates	Estimate	Standard Error	Z-value	*P*-value	Residual Heterogeneity *P*-value
Primary IVM	Volume	-16.90	2452.43	-0.01	0.99	1.00
	Marginal Dose	0.06	0.60	0.11	0.91	1.00
	Age	-0.03	0.10	0.30	0.77	1.00
	Publication Year	0.04	0.14	0.30	0.76	0.61
Total IVM	Volume	2.07	0.97	2.13	0.03	1.00
	Marginal Dose	0.19	0.09	2.01	0.04	1.00
	Age	-0.03	0.09	-0.33	0.74	1.00
	Publication Year	0.01	0.24	0.04	0.97	0.88
Perifocal Edema	Volume	1.05	1.32	0.80	0.42	0.64
	Marginal Dose	0.23	0.12	1.84	0.07	0.28
	Age	0.10	0.06	1.64	0.10	0.75
	Publication Year	0.25	0.26	0.94	0.35	0.45

### Post-SRS perifocal edema

**Fig. 4:**
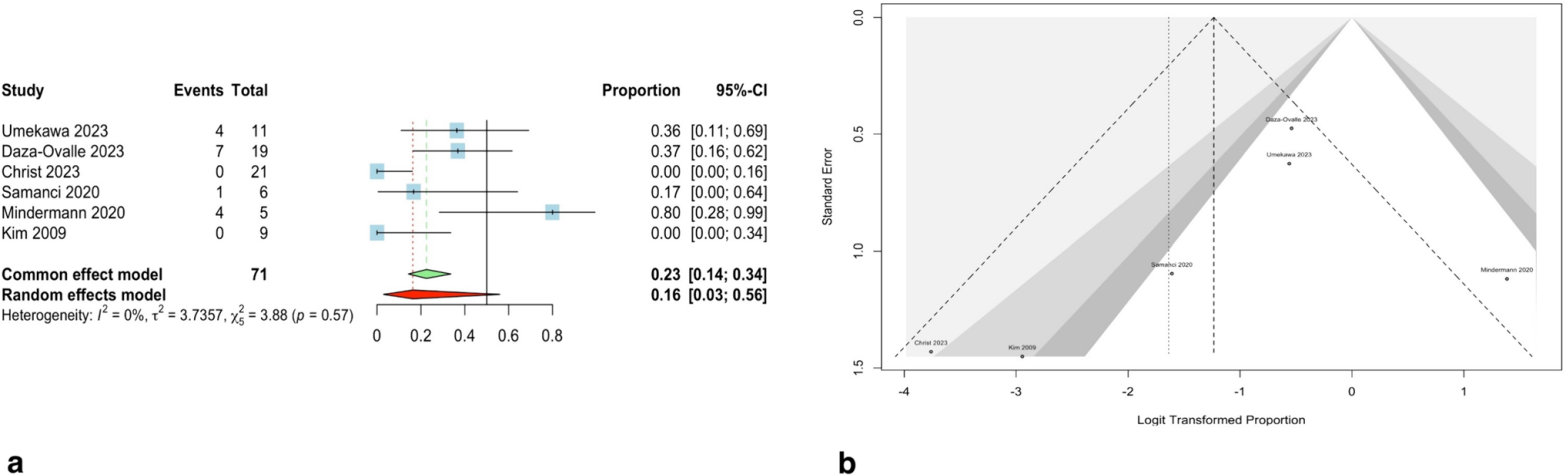
**a** The forest plot for the meta-analysis shows 16% perifocal edema following SRS. Also, the heterogeneity of studies was not significant (*P* = 0.57). **b** The funnel plot indicates no significant asymmetry, suggesting no publication bias among the studies

The meta-analysis for the occurrence of post-SRS edema was performed on a total 71 patients who underwent SRS. Overall, 16 patients (0.16; 95% CI, 0.03–0.56) experienced perifocal edema with 7 symptomatic cases. The random-effect meta-analysis showed an overall proportion of 0.44 for symptomatic edema cases (95% CI, 0.22–0.68). The forest plot and funnel plot are depicted in Fig. 4. The Egger’s linear regression test for publication bias was not significant (*P* = 0.32). The heterogeneity of the studies was low (*Q*: 3.88; *P*-value: 0.57, tau^2^: 3.74, and *I*^2^: 0%). Leave-one-out analysis found no outlier study. The influence diagnostics of the studies are depicted in Supplementary Fig. 4. A meta-regression analysis was conducted to assess the impact of median tumor volume, mean participant age, median radiation dosage, and year of publication on the occurrence of post-SRS edema, which were found insignificant with *P*-values of 0.42, 0.10, 0.07, and 0.35 respectively. Table 4 shows a summary of meta-regressions.

### Progression-free survival individual participant analysis

**Fig. 5:**
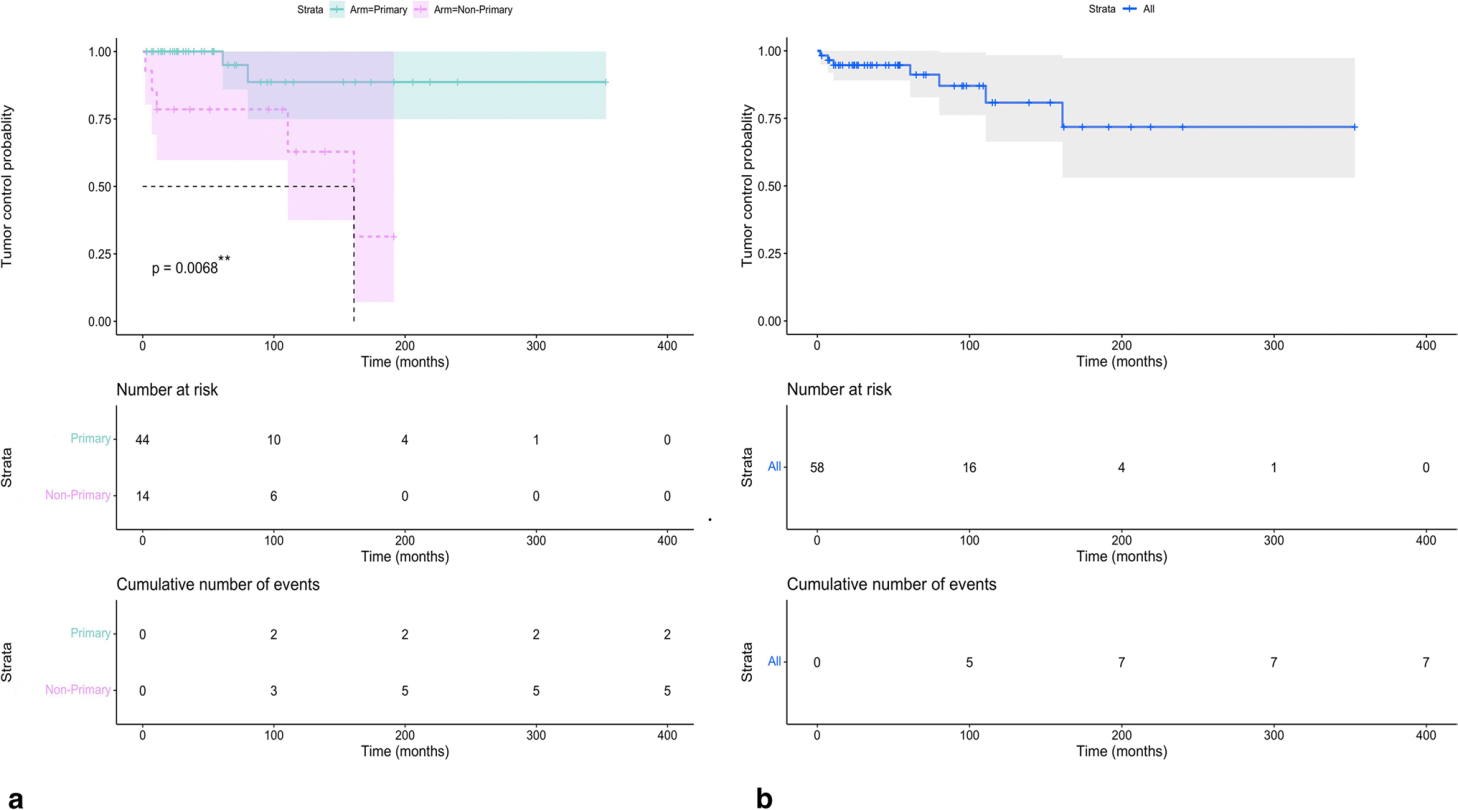
The Kaplan–Meier survival curves of patients. **a** The tumor control probability for primary and non-primary SRS for IVM has been shown. Also, a comparison between the two groups has been conducted, which showed a significant difference (*P* < 0.01). **b** The tumor control probability for all SRS procedures in IV meningioma has been shown

Individual data for tumor control of SRS in IVM was retrieved from 9 studies. A total of 58 tumors–follow-ups were available. A total 7 events occurred in a maximum of 353 months of follow-up. The PFS was 94.70% at 2 years’ follow-up. Two events occurred in the primary SRS group through an overall 44 tumors, and 5 events occurred in the non-primary SRS group through an overall 14 tumors. The log-rank test demonstrated a significantly higher PFS in primary compared to adjuvant SRS therapy (*P* < 0.01). The Kaplan–Meier survival curves are depicted in Fig. 5.

## Discussion

This study presents the first meta-analysis of the efficacy of SRS in IVM, including data from a total of 101 IVM tumors treated in 94 patients, regardless of whether the treatment was primary, adjuvant, or salvage. The results of this study show that 87% and 66% of patients treated with SRS achieved successful tumor control and regression at follow-up, respectively. In patients who received SRS as initial therapy, the tumor control rate was even higher at 92%. During the two-year follow-up period, the PFS rate of patients was 94.7%. The analysis showed that patients who had initially received SRS had a significantly better PFS. However, no correlation was found between the age of the patients and the size of the tumor. The study also found that a higher marginal dose and larger tumor size were associated with better control by SRS. Cerebral edema occurred in about 16% of patients after SRS, of which only 11% required surgical intervention, while the remaining cases were transient and treated conservatively. No significant correlation was found between the marginal dose, the age of the patients, the size of the lesion, and the occurrence of cerebral edema.

While resection can be an effective cure for meningiomas, it is not always the best option for patients who do not want to undergo resection or cannot tolerate general anesthesia [35]. In addition, depending on the location of the IVM, resection does not always result in complete removal of the tumor, or access to the tumor may involve traversing important fiber tracks and neural nodes [7, 13]. Studies have shown that subtotal resection occurs in 7% to 16% of cases, and recurrence of meningiomas occurs up to 28%. A study by Chen et al. found that 13% of surgical treated patients suffered mild to moderate complications, 19% severe complications, and 2% died [7]. The recurrence of patients who have undergone surgery has been found to be dependent on factors such as subtotal resection and a WHO grade other than I [7].

With the advent of SRS, the minimally invasive treatment of meningiomas, including IVM, has become more common [26]. A study conducted by Marchetti et al. examined the efficacy of SRS in the treatment of IVM. The study showed that SRS resulted in local control in 85% to 100% of patients during the 5-year follow-up period, with 74% to 99% of patients achieving PFS between 4 and 5 years. These results highlight the potential of SRS in the treatment of IVM and suggest that it could be a viable treatment option. Although our study mostly comprises WHO class I meningiomas, it also contains tumors from other classes, unlike the study mentioned [26]. In our meta-analysis of SRS for IVM, we found that larger tumor size was associated with better tumor control. However, it is worth noting that the tumors included in our analysis were small, and the median tumor volume of all included studies was less than 10 ml. Previous studies have indicated that tumors larger than 10 ml have worse outcomes following SRS [44]. Given the low number of patients in our meta-analysis, further studies are necessary to confirm these results, especially for tumors with a volume smaller than 10 ml.

In cases where tumors are found incidentally in asymptomatic patients, various treatment options are available, including resection, SRS, or a wait-and-see approach without taking any action. However, in these patients, SRS or follow-up is preferred to surgical treatment. The Daza-Ovale study found that SRS produced better results when performed earlier [9]. Moreover, resection seems to cause even more adverse events in asymptomatic patients than in symptomatic patients [34].

One complication associated with SRS is adverse radiation effects that can lead to edema among other sequalae. This complication is more likely to occur if the tumor is larger, certain tumor locations are affected, and the SRS is more than 5 Gy per fraction [19, 26]. The incidence rate of cerebral edema associated with SRS in our study was similar to that of cerebral edema in para-sagittal regions, which has been reported to be between 10–40% [5]. In fact, it seems that despite IVM being considered a different identity from other meningiomas, the response to SRS treatment in them is not much different from other meningiomas, and its complications are comparable to meningiomas in other locations. Radiation necrosis is another important complication that could occur following SRS, while it was not reported through the included studies [23, 47].

Hydrocephalus is one of the expected manifestations of IVM, specially in 3rd and 4th ventricles. Chen et al. [6] and Daze-Ovalle et al. [10] reported ventriculoperitoneal (VP) shunt placement to resolve hydrocephalus before SRS (one case each). Hydrocephalus could be a complication following intraventricular interventions. However, there has been no report of hydrocephalus following SRS in the included studies [8, 20, 41], except for Liu et al. [23], who reported the development of entrapment of the temporal horn as a type of focal hydrocephalus that needs further intervention. Moreover, Kim et al. [20] had one case with WHO grade II IVM who had recurrence after 7 months and died 1 month following fractionated radiotherapy due to VP shunt failure.

It is important to point out the limitations of this study, which include a small sample size of patients. In addition, the lack of results based on the WHO classification may limit the applicability of the results to all classes of meningioma. Another factor that should be considered is the location of the tumor, as accessibility may vary depending on location. In addition, the lack of histologic results for all patients is a shortcoming that affects the reliability of the results of the study. It is likely that the vast majority of the IV meningiomas in this study were grade I meningiomas; the results herein may not generalize to higher-grade meningiomas or other tumor types that are intraventricular. Moreover, future studies could also address the comparison between SRS, surgery, and hypofractionated radiosurgery. In order to strengthen the validity of the results, further investigations should be carried out. Further studies are also advisable to determine the optimal threshold dose. While a dose of 12–15 Gy is currently considered appropriate for grade I meningiomas, higher doses may be beneficial in recurrent cases [26].

## Conclusion

This study demonstrates high rates of tumor control with low complications for IVM when treated with SRS, regardless of previous treatment. SRS appears to be a promising alternative first-line treatment for asymptomatic IV meningiomas.

## Supplementary Information

Below is the link to the electronic supplementary material.

Supplementary file1 (DOCX 313 KB)

Supplementary file2 (DOCX 323 KB)

Supplementary file3 (DOCX 111 KB)

Supplementary file4 (DOCX 318 KB)

## Data Availability

The data presented in this study is available on reasonable request to the corresponding author.

## Abbreviations & acronyms

IVM: Intraventricular meningioma
SRS: Stereotactic radiosurgery
IPD: Individual patient data
PFS: Progression-free survival
PRISMA: Preferred Recording Items for Systematic Review and Meta-Analysis
JBI: Joanna Briggs Institute
VP: Ventriculoperitoneal

## Code Availability

The code used for analysis in this study is available at a reasonable request to the corresponding author.
