# Supplementary figures and images for: Stereotactic radiosurgery for intraventricular meningioma: a systematic review and meta-analysis
Source: Acta Neurochir (Wien). 2024 Jul 9;166(1):286. doi: 10.1007/s00701-024-06185-w (PMC11233396; doi:10.1007/s00701-024-06185-w)

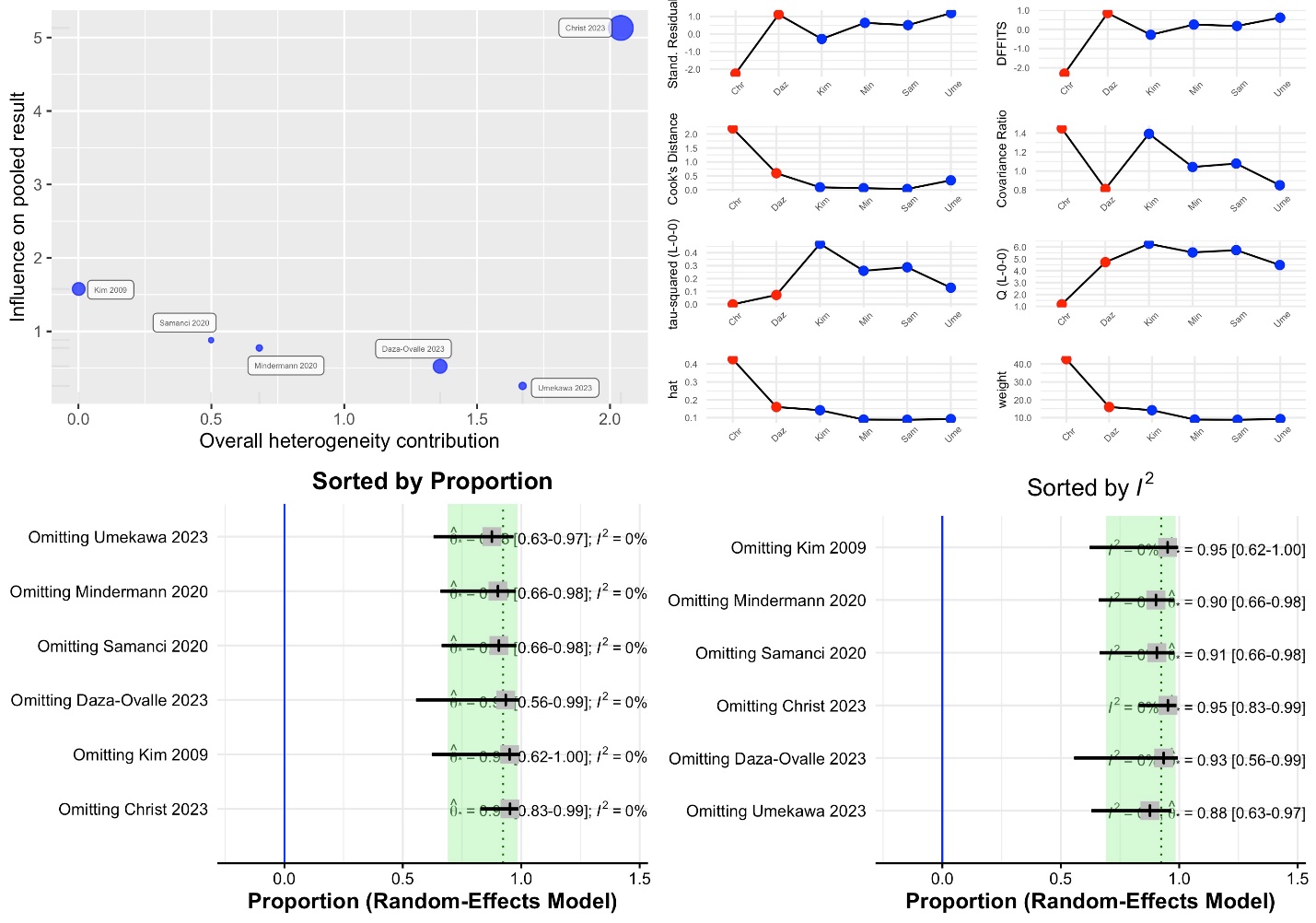


**Fig.1** Influence diagnostics of primary tumor progression have been shown.

Supplement: Supplementary file 1 — Supplementary file1 (DOCX 313 KB) [file 701_2024_6185_MOESM1_ESM.docx]

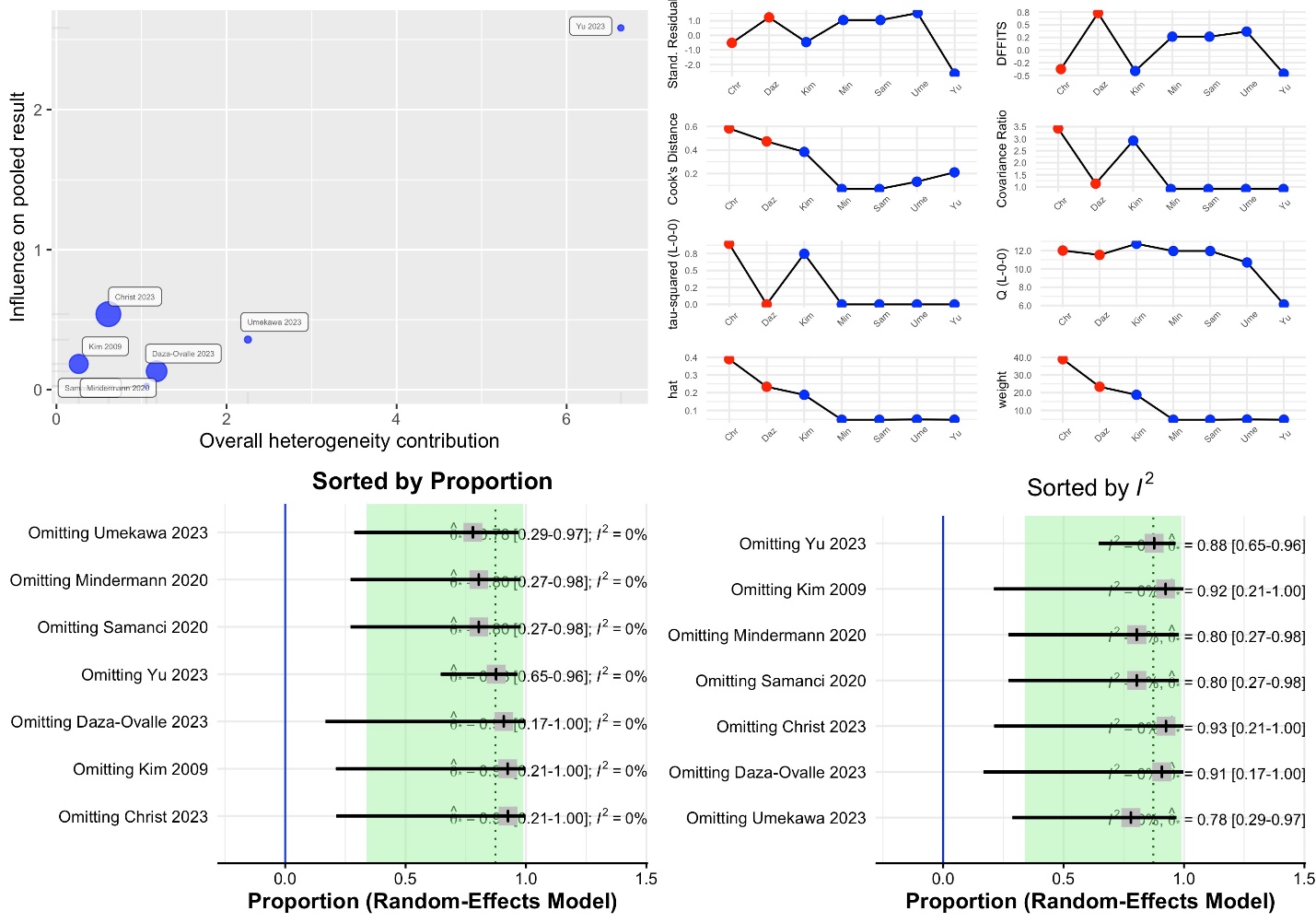


**Fig. 2** Influence diagnostics of progression of all IVMs have been shown.

Supplement: Supplementary file 2 — Supplementary file2 (DOCX 323 KB) [file 701_2024_6185_MOESM2_ESM.docx]

**
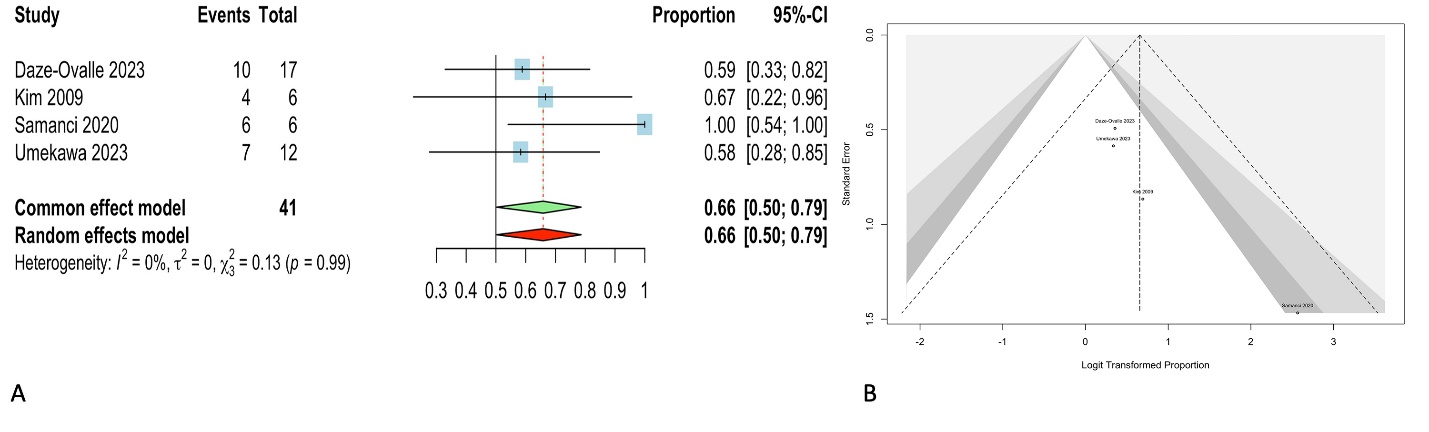
Fig 3.** The forest plot and funnel plot of regressed tumors

Supplement: Supplementary file 3 — Supplementary file3 (DOCX 111 KB) [file 701_2024_6185_MOESM3_ESM.docx]

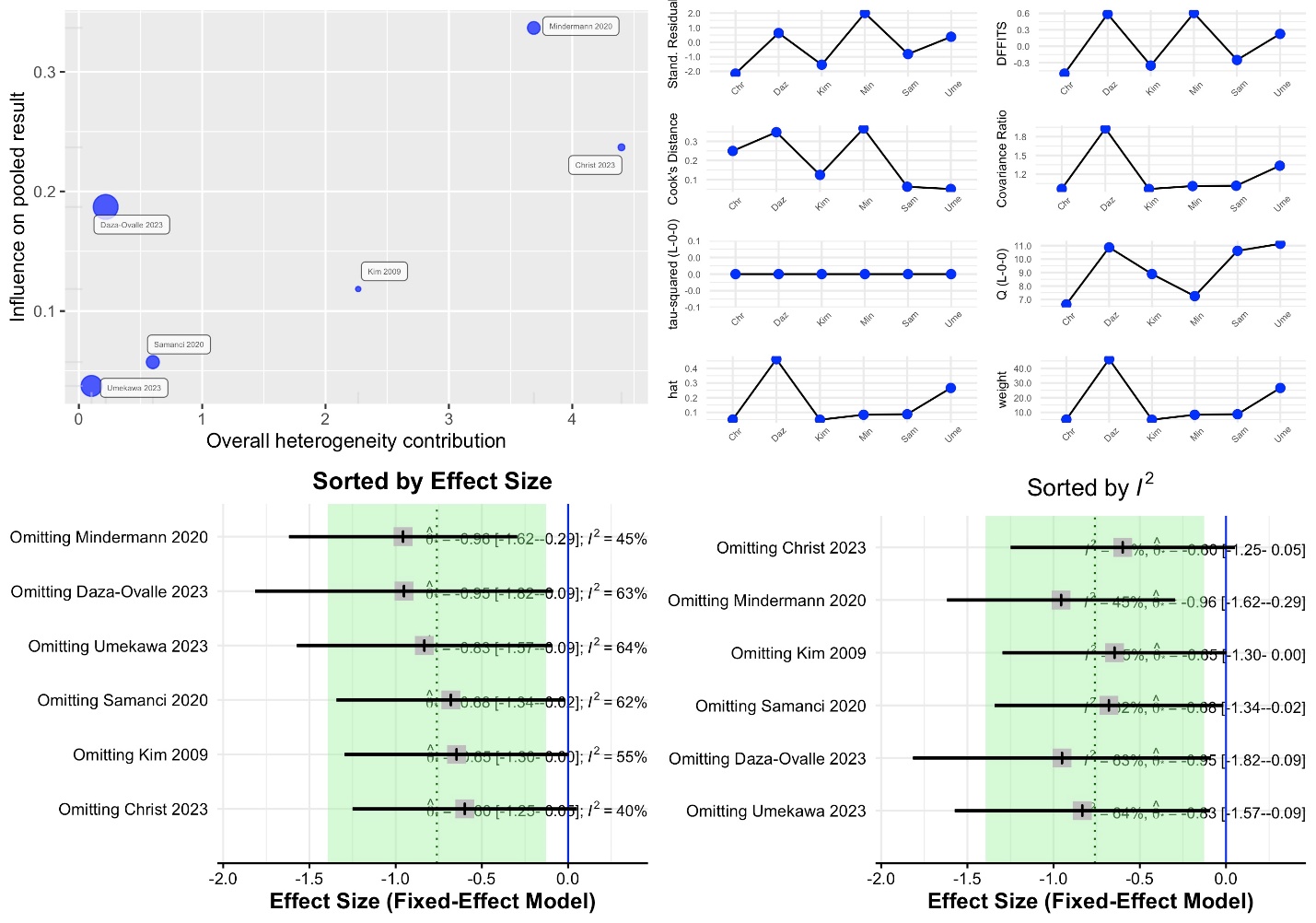


**Fig. 4** Influence diagnostics of all post SRS perifocal edema have been shown.

Supplement: Supplementary file 4 — Supplementary file4 (DOCX 318 KB) [file 701_2024_6185_MOESM4_ESM.docx]
